# The trend of opioid prescriptions among cancer patients in a tertiary hospital: A multimethod quantitative study

**DOI:** 10.3389/fonc.2023.1138169

**Published:** 2023-04-11

**Authors:** Pawita Limsomwong, Thammasin Ingviya, Orapan Fumaneeshoat

**Affiliations:** ^1^ Department of Family and Preventive Medicine, Prince of Songkla University, Songkhla, Thailand; ^2^ Division of Digital Innovation and Data Analytics, Faculty of Medicine, Prince of Songkla University, Songkhla, Thailand

**Keywords:** cancer, opioids, oral morphine equivalent, pain, trend, Thailand

## Abstract

**Introduction:**

Pain is a major symptom in cancer patients. World Health Organization recommends opioids as the main analgesic agent. Few studies have examined the amount of opioid uses in cancer patients in Southeast Asia, however, none of them have examined the factors associated with the amount of opioid uses which were lower than required.

**Objectives:**

To assess the trends and factors associated with opioid prescriptions for cancer patients in Songklanagarind Hospital, the largest referral center in Southern Thailand.

**Design:**

Multi-method quantitative study.

**Methods:**

We reviewed the electronic medical records of 20,192, outpatients aged ≥18 years diagnosed with cancer between 2016 and 2020 who received opiod prescriptions. Oral morphine equivalents (OME) were calculated using the standard conversion factors and the OME trend during the study period was assessed by a generalized additive model. Factors affecting the morphine equivalent daily dose (MEDD) were assessed using multiple linear regression with a generalized estimating equation.

**Results:**

The mean overall MEDD for all study patients was 27.8 ± 21.9 mg per day per patient. The bone and articular cartilage cancer patients had the highest MEDD. For every 5-year increase in the duration of cancer, the MEDD increased by 0.02 (95% confidence interval [CI]: 0.01 - 0.04). Patients with stage 4 cancer received a higher average MEDD of 4.04 (95% CI: 0.30-7.62) as compared to those with stage 1 cancer. Patients with bone metastasis received a average higher MEDD of 4.03 (95% CI: 0.82-7.19) compared to those without. Age was inversely associated with the MEDD. Patients aged 42-58, 59-75 and >76years old received MEDDs of 4.73 (95% CI: 2.31-7.15), 6.12 (95% CI: 3.66-8.59) and 8.59 (95% CI: 6.09-11.09) compared with those aged 18-42 years old. Brain metastasis was inversely associated with MEDD of 4.49 (95% CI: 0.61-8.37) compared to those without.

**Conclusion:**

Opioid use in cancer patients in this study is lower than the average global usage. Promoting opioid prescriptions for pain management through medical education can help doctors overcome opiophobia.

## Introduction

Opioids were listed as one of the “essential medicines in palliative care” in 2007 by the International Association of Hospice and Palliative Care (1). Thus, assessing the uses of opioids can infer the appropriateness of related palliative care practice.

Opioids are listed as the treatment of choice in the World Health Organization (WHO) step-ladder pattern for controlling moderate to severe pain (2). Pain is a common concern in cancer patients. 33% of cancer patients report significant pain at least once after their diagnosis (3). In the past decades, cancer treatments have become more advanced, and cancer survival rates have increased accordingly. Therefore, cancer patients may have a higher chance of developing cancer-related pain in their prolonged lives post-treatment.

Opioid use is an important indicator of a nation’s progress in dealing with cancer pain (4, 5). The factorsconcerning opioid use in Western countries differ from those in Asian countries. Addiction and abuse are major concerns in Western countries (6), where opioid usage is much higher than in Asian countries. Low opioid use in Asian countries is associated with widespread reports of inadequate pain control, especially in cancer patients (7).

The morphine equivalent daily dose (MEDD) is a common measurement that is widely used to estimate and compare opioid usage among countries and settings (8). Decreasing trends of MEDD in the US and European countries have been reported in various studies since opioid restriction acts were initiated in 2014 (9), while the MEDD trends in Asian countries have been increasing (10, 11) but are currently at levels that are much lower than in the US and UK.

Since the enactment of the Narcotics Act in 1979, the Thai government has classified all opioids and opioid-containing products as category two narcotics, which means that whoever unauthorizedly possesses, disposes, or uses opioids shall be fined or imprisoned (12). Opioid usage was legally allowed for research or medical practice (13). Medical purpose usage was controlled and monitored by the Thai Ministry of Public Health (14). In 2014, 35 years after the enactment of the narcotics acts, the Thai Ministry of Public Health enacted the Palliative Care Policy to increase the quality and coverage of palliative care services. To help overcome the limited use of opioids by healthcare workers who feared being fined or imprisoned under the narcotics act, the palliative care policy encouraged the use and promoted the accessibility of opioid medication for palliative patients requiring pain control. However, the effectiveness of this policy has not yet been examined.

Although changes in regulations affecting opioid use in the US and Europe were designed to decrease opioid use, the Thai palliative care policy hope to increase its use. Additionally, to promote the appropriate use of opioids for pain control, it is important to assess the factors affecting opioid prescriptions in cancer patients. Various studies have shown that certain factors, including older age, may limit the use of opioids for pain control (9, 15).

In our review, we found only one study associated with opioid consumption in Southeast Asia, which was performed in Malaysia (10) using a defined daily dose per 1,000 populations per day. However, this study was limited due to the use of inappropriate divisors. The optimal method for calculating opioid use may be the MEDD, which can increase the validity of how opioid use is assessed in the at-risk population. We hypothesized that opioid use in Thailand was still low. Therefore, this study aimed to assess the trend and current opioid usage in cancer patients using a database from the largest tertiary hospital in Southern Thailand and the factors associated with the MEDD of individual opioid prescriptions to guide how and which subgroups of cancer patients should have opioid use promotion strategies implemented.

## Methods

### Study design and setting

The study had a multimethod quantitative design and was conducted based on the electronic medical records of patients who visited Songklanagarind Hospital, the largest cancer center in Southern Thailand. The first part of the study was an ecological design in which the MEDD was calculated as a yearly summary by primary cancer site to assess the MEDD trend over the 5-year study period (2016–2020). The second part of the study used a longitudinal analysis design, in which the MEDDs for individual prescriptions were calculated to assess the factors associated with individual opioid prescriptions, accounting for the collinearity dependency of the data in the individual patient data.

### Data source

The cancer diagnoses were confirmed using the Southern Thailand Cancer Registry database. The primary cancer sites were classified using the International Classification of Diseases and Related Health Problems, 10th Revision, Thai Modification (ICD-10 TM) (16) and the International Classification of Diseases for Oncology (ICD-O) (17). The experts responsible for the cancer registry confirmed all cancer diagnoses by reviewing pathological and/or imaging reports of the patients.

To calculate the MEDD, outpatient prescription data during the 5-year study period were extracted from the Hospital Information System (HIS) of Songklanagarind Hospital. We limited the study period from 2016 to 2020, as in 2021 and 2022 the coronavirus disease outbreak might have affected the opioid usage. Other data extracted included pain score, age, sex, religion, primary cancer type based on the ICD-10-TM; and information on opioid administration, including the generic name, dosage, route of administration, and treatment duration.

### Participant selection

Patients aged ≥18 years on the day of their cancer diagnosis who had received any medication during the 5-year study period were included. The patients were divided into four age groups using quartiles. The primary cancer sites were divided into 14 groups by ICD-10 codes based on their broad grouping behaviors.

Both transdermal and oral opioids were included in the MEDD calculations. The only transdermal opioid available in the hospital during the study period was fentanyl. The available oral opioids included tramadol, codeine phosphate, morphine, and oxycodone.

### Oral morphine equivalent and MEDD calculations

To calculate the MEDD and OME were calculated. All oral and transdermal opioids in the same prescription for each patient on one day were converted using the standard OME equation: OME = strength per unit × [number of units/prescriptions] × MEs conversion factor. The conversion factors were retrieved from the Centers for Disease Control and Prevention (18). In the case of dosages prescribed for breakthrough pain, based on the opinion of pain management experts, half of the maximum possible dosage was applied in the calculation. The MEDD of each prescription was then calculated by dividing the OME per prescription by the intake duration (in days) of the prescribed drug.

The outcome of the first part of the study was the trend during the 5-year study period in yearly opioid use. The total sum of OME per patient per day was calculated by the primary cancer site, which represents the total amount of opioids prescribed for each primary cancer and compared across years and time periods (early two years [2017–2018] and final 3 years [2019–2021]). The mean total OME per patient per day was calculated from the total annual OME divided by the number of patients and 365.25 days within the corresponding year. The second part of the study assessed the factors associated with the MEDD. MEDD per prescription was used for the analysis.

### Statistical analysis

All analyses were performed using the R program (version 4.1.2; R Core Team, Austria) on the Jupyter Notebook on a server supported by the Division of Digital Innovation and Data Analytics, Faculty of Medicine, Prince of Songkla University. The patient characteristics were analyzed using numbers and corresponding percentages. The means with standard deviations and medians with interquartile ranges of the total sum of the OME per patient per day and MEDD by primary cancer type were calculated and compared. For the first part of the study, significant changes in the trend of the MEDD were assessed using a generalized additive model (GAM) accounting for year of prescription, age, sex and primary cancer site. For the second part of the study with a longitudinal design, the factors affecting the MEDD of individual prescriptions within and between patients were assessed using multiple linear regression with a generalized estimating equation (GEE) adjusted for covariates including duration of cancer, year of prescription, age, religion, primary cancer site, stage of cancer, metastases, and pain score. Statistical significance was set at p<0.05.

### Research ethics and patient consent

The study was approved by the appropriate ethics committee. The need for informed consent was waived due to the retrospective nature of the study.

## Results

**Figure 1:**
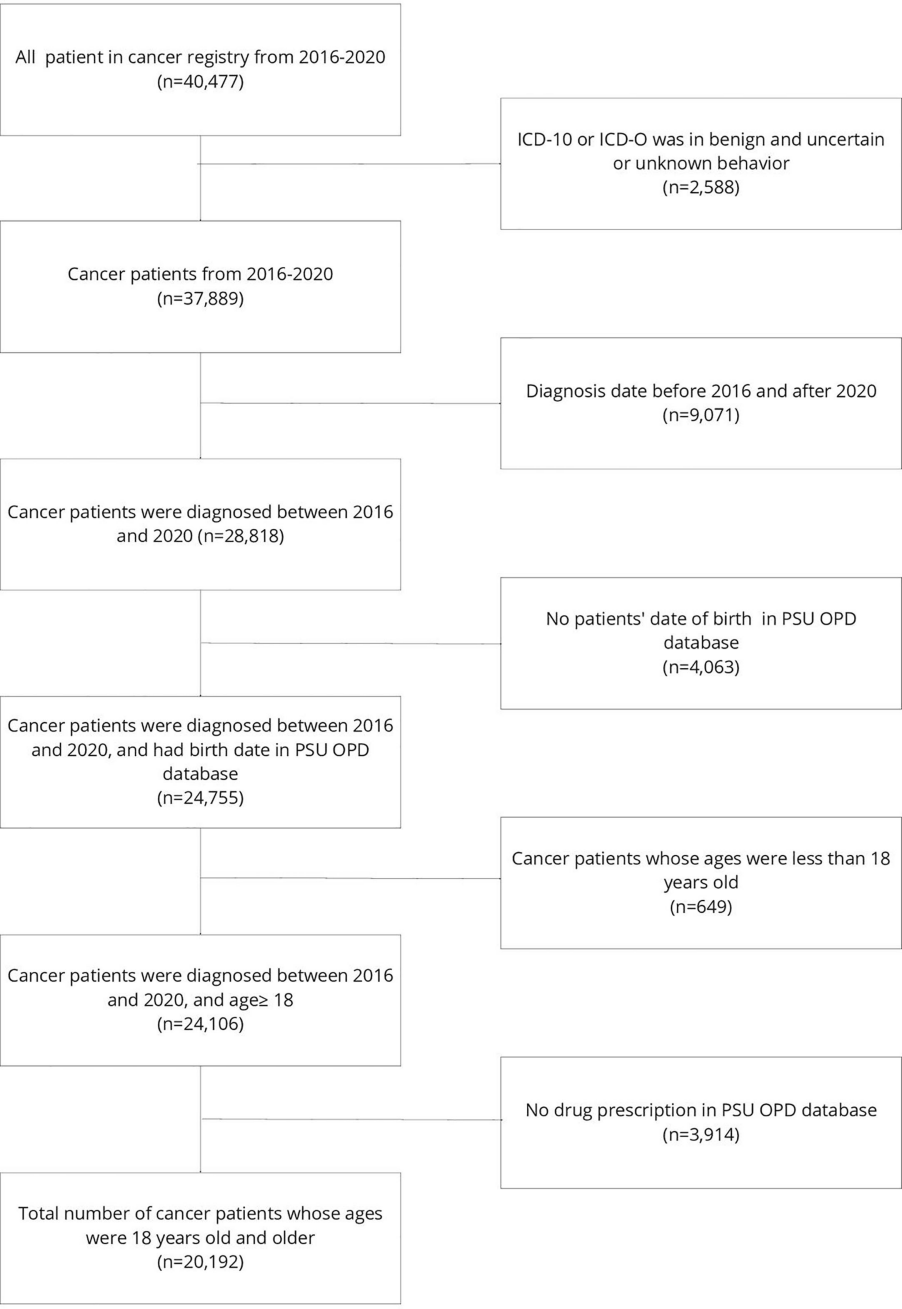
Participant flow chart.

A total of 40,477 patients were included in the cancer registry of Songklanagarind Hospital during the study period. We excluded 9,071 patients whose diagnosis date was before January 2016 or after December 2020, 4,063 patients with no date of birth, and 3,914 patients whose drug prescriptions were not included in the PSU OPD database, leaving 20,192 patients included in the final analysis. The study flowchart is presented in **Figure 1**. Of the 20,192 eligible patients, most (75.9%) were aged between 42 and 76 years old. Buddhism was the most common religion among the patients.

### Trend in opioid prescriptions

The average dose of an opioid per patient per day in Songklanagarind Hospital in the cancer patients during the study period 2016-2020 was 1.41 ± 0.43 to 1.57 ± 0.64 mg per day per patient. Male cancer patients received more opioids than female patients throughout the study period. Additionally, the cancers with the highest mean OME per day per patient were bone and articular cartilage cancers as shown in **Table 1**.

**Table 1:** Characteristic of cancer patients visiting the outpatient department and the opioid consumption per patient per day per year during 2016–2020 (N=20,192).

Factor		Total	Opioid consumption per patient per day by year (mg)					P-value
			2016	2017	2018	2019	2020	
All		20,192 (100)	1.53 (0.49)	1.49 (0.53)	1.57 (0.60)	1.56 (0.64)	1.41 (0.57)	<0.001
Age	18-41	2,544 (12.6)	0.93 (0.33)	1.38 (0.80)	1.38 (0.73)	1.57 (0.78)	1.39 (0.92)	0.005
	42-58	7,457 (36.9)	1.86 (0.51)	1.66 (0.56)	1.71 (0.66)	1.65 (0.67)	1.43 (0.54)	<0.001
	59-75	7,868 (39.0)	1.46 (0.50)	1.36 (0.39)	1.58 (0.52)	1.57 (0.60)	1.51 (0.51)	<0.001
	>76	2,323 (11.5)	1.37 (0.46)	1.49 (0.48)	1.27 (0.40)	1.27 (0.46)	0.97 (0.39)	<0.001
Sex	Female	11,001 (54.4)	1.16 (0.45)	0.99 (0.45)	0.86 (0.40)	1.07 (0.46)	1.05 (0.51)	<0.001
	Male	9,191 (45.6)	1.95 (0.52)	2.06 (0.61)	2.47 (0.76)	2.23 (0.81)	1.92 (0.64)	<0.001
Primary cancer site	Bones and articular cartilage	75 (0.4)	5.51 (0.74)	2.00 (0.55)	5.33 (1.35)	8.47 (2.69)	13.38 (4.43)	0.601
	Breast	2,485 (12.3)	0.56 (0.29)	0.76 (0.39)	0.66 (0.32)	0.80 (0.37)	0.78 (0.38)	0.712
	Endocrine system	1,355 (6.7)	0.06 (0.13)	0.07 (0.17)	0.24 (0.53)	0.23 (0.43)	0.20 (0.37)	0.140
	Eye, brain, and other nervous system	239 (1.2)	0.22 (0.15)	0.30 (0.13)	0.31 (0.19)	0.16 (0.14)	0.25 (0.23)	0.096
	Gastrointestinal	4,823 (23.9)	1.46 (0.52)	1.29 (0.56)	1.44 (0.58)	1.69 (0.70)	1.22 (0.46)	<0.001
	Gynecological	2,964 (14.6)	0.58 (0.25)	0.55 (0.30)	0.58 (0.35)	0.71 (0.38)	0.79 (0.38)	0.693
	Haematological	1,183 (5.9)	0.69 (0.40)	1.22 (0.69)	0.97 (0.56)	0.85 (0.53)	0.63 (0.34)	0.216
	Head and neck	2,014 (10.0)	3.33 (0.60)	3.09 (0.62)	3.58 (0.86)	3.05 (0.71)	2.9 (0.64)	<0.001
	Male genitals	1,001 (4.9)	0.38 (0.25)	0.89 (0.44)	0.73 (0.41)	0.61 (0.31)	0.97 (0.47)	0.118
	Malignant neoplasms of ill-defined, secondary, and unspecified sites	204 (1.0)	3.86 (0.89)	3.53 (0.69)	4.28 (1.25)	2.93 (0.51)	3.82 (0.70)	0.273
	Mesothelial and soft tissue	293 (1.5)	1.53 (0.64)	1.38 (0.39)	2.16 (0.48)	2.02 (0.53)	2.21 (0.60)	0.403
	Respiratory and intrathoracic organs	2,535 (12.5)	3.52 (0.60)	3.36 (0.60)	3.85 (0.70)	4.32 (0.93)	3.92 (0.75)	0.001
	Skin	483 (2.4)	0.67 (0.65)	0.82 (0.56)	0.65 (0.40)	0.66 (0.32)	0.65 (0.32)	0.975
	Urinary tract	538 (2.7)	0.51 (0.28)	1.70 (0.68)	1.76 (0.62)	1.35 (0.49)	1.77 (0.67)	0.942

Overall, the mean MEDD was 27.8 ± 21.9 mg per day per patient during the 5-year study period. In the early years (2016–2018), the mean MEDD was 26.8 ± 19.7, which slightly increased to 29.0 ± 24.0 during 2018–2020. The five cancers with the highest mean MEDDs were bone and articular cartilage cancer; oral cavity and pharynx cancer; malignant neoplasms of ill-defined, secondary, and unspecified sites; mesothelial and soft tissue cancer; and respiratory and intrathoracic organ cancers. The mean MEDD was significant higher in patients with high cancer stages and metastases of the bone, brain, lung, liver, and peritoneum (**Table 2**).

**Table 2:** Distribution of MEDD by cancer type, staging, and metastasis during 2016–2020 (N= 7,424).

Variable		Number of Patients N (%)			Overall (2016-2020)	Early Two years (2016-2017)	Recent years (2018-2020)	P-value
All			Mean ±SD	27.8±21.9	26.8±19.7	29.0±24.0	<0.001
			Median (IQR)	30.0 (15,30)	30.0 (15,30)	30.0 (15,30)	0.003
Age		18-41		Mean ±SD	32.2±27.9	30.2±25.0	34.2±30.3	<0.001
			Median (IQR)	30.0 (15,30)	30.0 (15,30)	30.0 (15,30)	<0.001
	42-58		Mean ±SD	28.7±22.3	27.5±19.8	30.1±24.7	<0.001
			Median (IQR)	30.0 (15,30)	30.0 (15,30)	30.0 (15,30)	0.048
	59-75		Mean ±SD	26.7±20.2	25.7±17.9	27.7±22.3	<0.001
			Median (IQR)	25.0 (15,30)	25.0 (15,30)	25.0 (15,30)	0.002
	>76		Mean ±SD	25.1±19.5	25.5±20.3	24.7±18.5	0.303
			Median (IQR)	25.0 (15,30)	25.0 (15,30)	15.0 (11.2,30)	<0.001
Sex	Female		Mean ±SD	26.9±22.7	25.8±21.1	28.0±24.0	<0.001
			Median (IQR)	25 (15,30)	20 (15,30)	25 (15,30)	<0.001
	Male		Mean ±SD	28.5±21.4	27.4±18.9	29.8±24.0	<0.001
			Median (IQR)	30 (15,30)	30 (15,30)	30 (15,30)	0.036
Primary Cancer Type							
Bones and articular cartilage	49 (0.7)		Mean ±SD	36.5±39.0	26.0±14.4	46.1±50.4	<0.001
			Median (IQR)	30.0 (15,30)	30.0 (15,30)	25.0 (15,30)	0.681
Breast	531 (7.2)		Mean ±SD	25.1±18.8	24.2±19.6	23.2±18.4	0.124
			Median (IQR)	15.0 (15,30)	15.0 (11.2,30)	20.0 (15,30)	0.012
Endocrine system	80 (1.8)		Mean ±SD	26.0±14.4	22.8±11.3	28.0±15.7	<0.001
			Median (IQR)	25.0 (15,30)	17.5 (15,30)	25.0 (15,30)	0.008
Eye, brain, and other nervous system	45 (0.7)		Mean ±SD	18.6±7.4	18.2+6.0	18.3±9.6	0.408
			Median (IQR)	15.0 (11.2,25)	15.0 (15,25)	25.0 (11.2,30)	0.866
Gastrointestinal	2,112 (28.4)		Mean ±SD	26.4±23.4	25.2±22.8	27.8±24.0	<0.001
			Median (IQR)	20.0 (15,30)	15.0 (15,30)	20.0 (15,30)	<0.001
Gynecological	767 (10.3)		Mean ±SD	25.7±18.6	25.4±17.5	25.9±19.6	0.549
			Median (IQR)	25.0 (15,30)	25.0 (15,30)	25.0 (15,30)	0.851
Eye, brain, and other nervous system	45 (0.7)		Mean ±SD	18.6±7.4	18.2+6.0	18.3±9.6	0.408
			Median (IQR)	15.0 (11.2,25)	15.0 (15,25)	25.0 (11.2,30)	0.866
Haematological	320 (4.3)		Mean ±SD	23.3±26.9	22.6±23.8	24.3±30.1	0.419
			Median (IQR)	15.0 (11.2,25)	15.0 (11.2,26.2)	15.0 (11.2,25)	0.983
Head and neck	1,369 (18.7)		Mean ±SD	31.7±19.8	30.7±17.3	33.1±22.9	<0.001
			Median (IQR)	30.0 (30,30)	30.0 (30,30)	30.0 (30,30)	0.282
Male genitals	231 (3.1)		Mean ±SD	22.4±15.1	22.2±12.6	22.8±16.8	0.695
			Median (IQR)	15.0 (15,30)	15.0 (15,30)	15.0 (11.2,30)	0.233
Malignant neoplasms of ill-defined, secondary, and unspecified sites	112 (1.5)		Mean ±SD	31.5±21.5	28.4±19.3	35.7±23.6	<0.001
			Median (IQR)	30.0 (15,30)	30.0 (15,30)	30.0 (20,42.5)	0.003
Mesothelial and soft tissue	141 (1.9)		Mean ±SD	30.3±25.5	24.7±17.3	36.1±30.9	<0.001
			Median (IQR)	25.0 (15,30)	20.0 (15,30)	30.0 (15,40)	<0.001
Respiratory and intrathoracic organs	1,344 (18.1)		Mean ±SD	28.5±23.0	27.0±19.7	30.2±26.3	<0.001
			Median (IQR)	25.0 (15,30)	25.0 (15,30)	30.0 (15,30)	0.017
Skin	130 (1.8)		Mean ±SD	26.4±17.8	24.0±15.7	28.2±19.2	0.026
			Median (IQR)	25.0 (15,30)	25.0 (15,30)	30.0 (15,30)	0.044
Urinary tract	193 (2.6)		Mean ±SD	26.5±21.6	26.0±19.4	28.8±23.1	0.089
			Median (IQR)	24.5 (15,30)	20.0 (15,30)	25.0 (15,30)	0.756
Stage							
Stage 1	282 (3.8)		Mean ±SD	24.4±23.6	23.1±14.2	25.4±28.8	0.169
			Median (IQR)	15.0 (15,30)	15.0 (15,30)	15.0 (11.2,30)	0.019
Stage 2	716 (9.6)		Mean ±SD	24.1±17.4	23.5±15.6	24.5±18.8	0.177
			Median (IQR)	15.0 (15,30)	15.0 (15,30)	15.0 (15,30)	0.400
Stage 3	1,481 (20.0)		Mean ±SD	26.6±19.7	26.1±18.9	27.0±20.4	0.099
			Median (IQR)	25.0 (15,30)	25.0 (15,30)	25.0 (15,30)	0.449
Stage 4	3,477 (46.8)		Mean ±SD	30.1±23.3	28.9±21.2	31.6±25.5	<0.001
			Median (IQR)	30.0 (15,30)	30.0 (15,30)	30.0 (15,30)	<0.001
Unknown stage	1,468 (19.8)		Mean ±SD	24.3±23.3	22.4±16.4	26.6±24.3	<0.001
			Median (IQR)	15.0 (15,30)	15.0 (11.2,30)	15.0 (15,30)	<0.001
Metastasis							
Bone	987 (13.3)		Mean ±SD	30.5±27.1	28.7±24.9	32.3±29.2	<0.001
			Median (IQR)	25.0 (15,30)	25.0 (15,30)	30.0 (15,30)	<0.001
Brain	238 (3.2)		Mean ±SD	25.7±18.2	24.2±17.1	27.4±19.2	0.004
			Median (IQR)	25.0 (15,30)	20.0 (15,30)	25.0 (15,30)	0.011
Liver	729 (9.8)		Mean ±SD	32.4±30.4	30.2±29.3	34.4±31.3	<0.001
			Median (IQR)	30.0 (15,30)	25.0 (15,30)	30.0 (15,30)	<0.001
Lung	990 (13.3)		Mean ±SD	29.4±25.7	26.7±21.3	32.1±29.3	<0.001
			Median (IQR)	25.0 (15,30)	20.0 (15,30)	30.0 (15,30)	<0.001
Lymph node	241 (3.2)		Mean ±SD	31.7±25.2	30.0±17.8	33.0±29.7	0.069
			Median (IQR)	30.0 (15,30)	30.0 (15,30)	30.0 (15,30)	0.717
Peritoneum	157 (2.1)		Mean ±SD	31.9±20.0	26.3±17.6	35.4±20.6	<0.001
			Median (IQR)	30.0 (15,40)	20.0 (15,30)	30.0 (20,55)	<0.001

**Figure 2:**
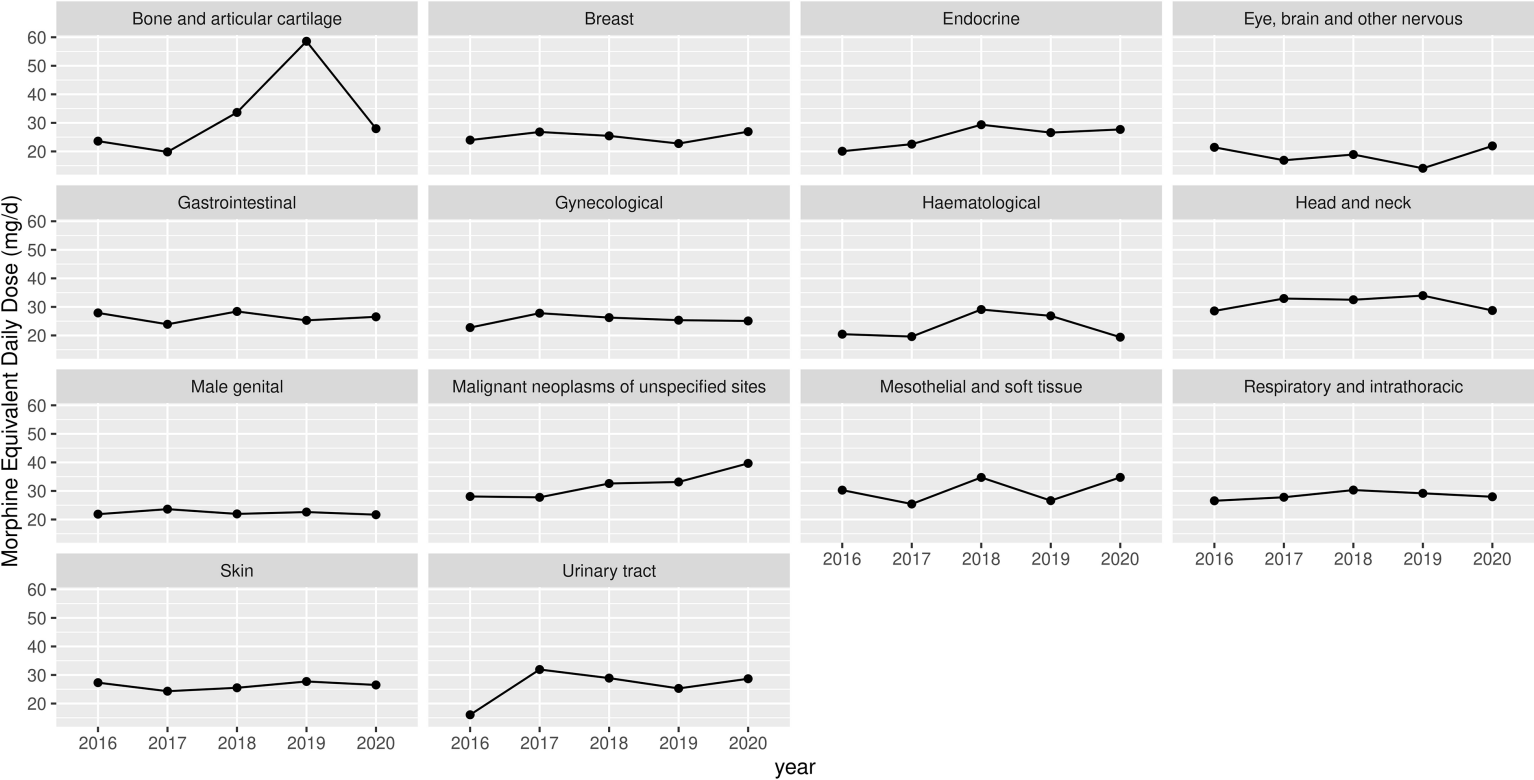
The usage of opioid (MEDD) over a period of 5 years.

The trends in MEDD differed significantly among the cancer types. The MEDD in bone cancer patients increased dramatically in 2019. The mean MEDDs were not significantly related to either year of prescription or sex. The trends and changes in MEDD by age and cancer type are shown in **Table 3** and **Figure 2**.

**Table 3:** Adjusted* morphine equivalent daily dose (MEDD) by age, sex, cancer type and calendar year from a generalized additive model (GAM).

Factor		Mean Diff(mg/d)	p-value
Calendar Year	2016	Ref	ref
	2017	1.27 (-1.36–3.90)	0.336
	2018	1.28 (-1.31–3.87)	0.323
	2019	1.49 (-1.07–4.06)	0245
	2020	1.32 (-1.31–3.96)	0.315
Age	18-41	Ref	Ref
	42-58	-1.04(-3.35–1.27)	0.366
	59-75	-2.33(-4.63–-0.03)	0.043
	>76	-4.74(-7.26–-2.21)	<0.001
Sex	Female	Ref	Ref
	Male	0.38(-1.36–2.13)	0.660
Primary Cancer Type	Bones and articular cartilage	Ref	Ref
	Breast	-5.97 (-11.74–-0.19)	0.039
	Endocrine system	-7.71 (-13.92–-1.49)	0.013
	Eye, brain, and other nervous system	-12.49 (-19.52–-5.47)	<0.001
	Gastrointestinal	-5.66 (-10.93–-0.39)	0.032
	Gynecological	-5.84 (-11.59–-0.10)	0.042
	Haematological	-9.10 (-14.50–-3.69)	<0.001
	Head and neck	-0.10 (-5.42–5.22)	0.969
	Male genitals	-8.43 (-14.72–-2.13)	0.007
	Malignant neoplasms of ill-defined, secondary, and unspecified sites	0.29 (-5.91–6.49)	0.925
	Mesothelial and soft tissue	-2.59 (-8.32–3.15)	0.368
	Respiratory and intrathoracic organs	-4.69 (-10.07–0.69)	0.081
	Skin	-3.16(-9.05–2.73)	0.284
	Urinary tract	-4.39 (-10.08–1.30)	0.123

*Adjusted for age, sex, the year of opioid prescription and cancer type.

**Table 4** presents the results from a multivariate linear regression model with GEE to explain duration of cancer, year of prescription, age, religion, primary cancer site, stage of cancer, metastases, and pain score. The study found that bone and articular cartilage cancer patients had the highest MEDD. For every 5 years increase in the duration of cancer, the MEDD increased by 0.02 (95% confidence interval [CI]: 0.01 - 0.04). Patients with stage 4 cancer received higher MEDD of 4.04 (95% CI: 0.30-7.62) as compared to those with stage 1 cancer. Patients with bone metastasis received generally higher MEDD of 4.03 (95% CI: 0.82-7.19) compared to those without. Age was inversely associated with the MEDD. Patients aged 42-59, 59-76 and 76 years old, received lower MEDD by 4.73 (95% CI: 2.31-7.15), 6.12 (95% CI: 3.66-8.59) and 8.59 (95% CI: 6.09-11.09), as compared with those aged 18-42 years old. Patients with brain metastasis received lower MEDD by 4.49 (95% CI: 0.61-8.37) mg than the other patients. Pain scores were positively associated with MEDD.

**Table 4:** Univariable and multivariate linear regression models of predictors of opioid use in cancer patients during the study period (N=7,424).

Factor		Univariable		Multivariable	
		MEDD diff (mg/d)	p-value	MEDD diff (mg/d)	p-value
Duration of cancer per 5-year increase		0.02 (0.01–0.03)	<0.001	0.02 (0.01–0.04)	<0.001
Year of prescription		-0.92 (0.92–0.93)	<0.001	0.11 (-0.38–0.59)	0.670
Age at prescription	18-41	ref	<0.001	ref	<0.001
	42-58	-3.24 (-5.53–-0.95)		-4.73 (-7.15–-2.31)	
	59-75	-5.02 (-7.30–-2.75)		-6.12 (-8.59–-3.66)	
	>76	-6.45 (-9.15–-3.75)		-8.59 (-11.09–-6.09)	
Sex	Female	ref	0.005	ref	0.581
	male	1.62 (0.48–2.74)		0.49 (-1.72–2.70)	
Religion	Buddhist	ref	0.271	ref	0.959
	Christian	2.81 (-1.27–6.88)		4.86 (1.23–8.49)	
	Islam	-0.84 (2.25–-0.58)		-0.10 (-1.74–1.55)	
	Others	1.01 (-11.57–13.60)		28.28 (23.76–32.79)	
Type of cancer	Bones and articular cartilage	ref	0.213	ref	<0.001
	Head and Neck	-3.11 (-13.17–6.95)		1.87 (-14.06–17.80)	
	Malignant neoplasms of ill-defined, secondary, and unspecified sites	-3.29 (-13.84–7.27)		-2.05 (-19.22–15.13)	
	Mesothelial and soft tissue	-4.73 (-16.22–6.76)		-2.06 (-19.21–15.09)	
	Respiratory and intrathoracic organs	-6.02 (-16.13–4.09)		0.15 (-15.87–16.17)	
	Urinary tract	-6.99 (-17.83–3.84)		-4.92 (-20.92–11.09)	
	Gastrointestinal	-8.39 (-18.45–1.70)		-4.12 (-19.96–11.72)	
	Skin	-8.55 (-18.90–1.79)		1.58 (-14.6–17.834)	
	Gynecological	-9.05 (-19.13–1.02)		-3.56 (-19.53–12.40)	
	Endocrine	-9.09 (-19.62–1.43)		-6.54 (-22.87–9.80)	
	Breast	-9.88 (-20.00–0.24)		-6.74 (-22.69–9.21)	
	Haematological	-12.33 (22.95–-1.72)		-3.3 (-21.52–14.89)	
	Male genitals	-12.11 (-22.29–-1.93)		-10.97 (-27.12–5.18)	
	Eye, brain, and other nervous system	-15.62 (-25.82–-5.41)		-10.64 (-26.64–5.37)	
Stage of cancer	Stage 1	Ref	<0.001	Ref	<0.001
	Stage 2	0.34 (-2.64–3.31)		-0.7 (-4.37–2.90)	
	Stage 3	2.63 (-0.21–5.47)		2.67 (-0.94–6.28)	
	Stage 4	6.58 (3.77–9.39)		4.04 (0.30–7.62)	
Bone metastasis	No		0.002		0.013
	Yes	3.48 (1.32–5.64)		4.03 (0.82–7.19)	
Brain metastasis	No	Ref	0.410	Ref	0.023
	Yes	-0.95 (-3.20–1.31)		-4.49 (-8.37–-0.61)	
Liver metastasis	No	Ref	0.001	Ref	0.173
	Yes	5.26 (2.27–8.26)		1.7 (-0.77–4.28)	
Lymph metastasis	No	Ref	0.024	Ref	0.099
	Yes	3.76 (0.50–7.03)		2.65 (-0.50–5.81)	
Peritoneum metastasis	No	Ref	0.043	Ref	0.079
	Yes	3.28 (0.11–6.46)		3.10 (-0.36–6.60)	
Pain score	0-3	Ref	0.001	Ref	0.001
	3-7	1.57 (0.56–2.58)		2.06 (0.86–3.26)	
	7-10	1.09 (0.05–2.13)		1.36 (0.2–2.47)	

Concomitant paracetamol prescriptions were inversely associated with MEDD (MEDD difference: -3.75; 95% CI: -4.47 to -3.03, P, <0.001), while concomitant anticonvulsant and antidepressant prescriptions were positively associated with MEDD per patient (MEDD difference: 8.38; 95% CI: 6.54 to 10.22, P, <0.001, and MEDD difference: 3.17; 95% CI: 2.34 to 3.99, P, <0.001, respectively), as shown in **Supplementary Table 1**.

## Discussion

### Main findings/results of the study

During the study period, all cancer patients in a university hospital in Thailand received a total OME of approximately 1.41-1.57 mg per day per patient. The MEDD per prescription was the highest in patients with bone and articular cartilage cancer. Even after adjusting for covariates, including age, duration of cancer, and staging, the MEDD of patients with bone and articular cartilage cancer was still higher than those of patients with other types of cancer. Overall, male cancer patients received higher dose of opioids than female patients throughout the study period. However, after adjusting for covariates including age, year of prescription, and primary cancer site, the difference in MEDD between male and female patients became not statistically significant. This might be partially explained by the fact that larger proportions of male patients were in advanced cancer stages and had been diagnosed with cancers with higher incidences and more severe pain, including bone and articular cartilage cancer, compared to female patients (**Supplementary Table 2**).

### What this study adds

In patients with bone and articular cartilage cancer, pain can be caused by either direct pressure from the tumor on the nerves surrounding the bone or various cytokines released around the area, including interleukin-1β, tumor necrosis factor α, interleukin-6, epidermal growth factor, and platelet-derived growth factor (19, 20).

The MEDD exhibited slightly increasing trend over the study period; however, the magnitude of the changes in MEDD was relatively small, especially when compared with the changes in MEDD in the US population. One study reported that the MEDD in the US decreased from 150 mg/day in 2008 to 83 mg/day in 2014 (9), while the overall MEDD in this study slightly increased from 26.8 mg/day during 2016–2018 to 29 mg/day during 2018–2020. This level is much lower than the recommended dosage limit of 90 mg/day and well under the limit of 100 mg/day, which is the normal level indicating an overdose (21).

According to a report from the Thai Food and Drug Association (FDA), the prescription of FDA-approved opioids modestly increased each year during 2012–2018, similar to the findings of this study (22). However, the utilization of opioids in Thailand was still much lower than the annual allocated quota. Opioid accessibility issues have been found with morphine IV, oral methadone, and oxycodone (22). Opioids have been classified as a category 2 narcotic since the enactment of the narcotics act in 1979 in Thailand (23). The Ministry of Public Health has been regulating the amount and limiting the possession of opioids since then. Although the Ministry of Public Health has been trying to promote opioid use in recent years by implementing a quality service coverage insurance plan that includes compensation for opioid availability in 2014 (24), increases in opioid use have been slight. In the university hospital where our study was performed, we established a pain clinic in 2015 to support this policy through consultations related to pain management. However, even with this policy and the pain clinic, the MEDD slightly increased over the 5-year period after the pain clinic was established.

Therefore, factors associated with MEDD were assessed in the second part of our analysis. Young age, years with cancer, advanced-stage cancer, bone metastasis, and adjuvant treatment were significant positive predictors of opioid prescription.

In terms of age, MEDD was highest in young adult patients, under 42 years of age. Previous studies have reported a decrease in opioid use in older patients (15, 25). However, the association between age and pain perception remains uncertain. Some studies have reported decreased pain perception in older patients due to the degeneration of neurological systems, while other studies have reported decreases in the pain threshold in older patients, thus increasing pain perception (26). “Opiophobia,” a term introduced in the 1980s, might explain this phenomenon. This refers to the fear that some physicians have that the use of opioids might lead a patient to become addicted to the drug or develop severe side effects (27). Morevover, fears of overdose resulting in deep sedation or respiratory depression are also widespread among healthcare providers and the general population (28, 29). A lack of appropriate education is one of the significant barriers for adequate opioid prescriptions for pain control (30). These concerns can lead to the under-prescription of pain-relieving drugs, leading to the undertreatment of severe pain, especially in elderly patients (31). Improved education on the safe and effective use of opioids might relieve those exaggerated risk perceptions resulting in opiophobia.

Longer duration and cancer stage were associated with increased MEDD in this study. This might be related to various factors, including increased pain caused by cancer progression, larger tumors, irritation of nerves and tissues around the tumors, distant metastases, or therapy (32).

Bone metastases were found to be associated with higher MEDDs. Bone is a connective tissue containing a large number of sensitive neurons in both the periosteum and the bone marrow which mediate acute and chronic bone pain (33). Bone metastasis can stimualte these neurons leading to an increase in pain severity, eventually resulting in an increase in opioid consumption to control pain. In one study 75% of cancer patients with bone metastases reported having bone pain (34).

All adjuvant analgesics, except for acetaminophen, were positively associated with the MEDDs in our study. The combination of anticonvulsant and antidepressant drugs has been reported to have a double benefit in relieving pain along with mood symptoms (35). These and other studies indicate that opioids are not the only drugs which can be used in the treatment of pain and that, at least pharmacologically, multimodal approaches are feasible (36). Although corticosteroids are commonly used as adjunctive therapies for pain relief in moderate to severe pain by inhibiting prostaglandin synthesis and reducing vascular permeability (37, 38), we found no such association n this study. In our study, acetaminophen use was inversely associated with MEDD. Although patients with low-level pain controllable with acetaminophen might not require opioids, these findings also imply that opioid use could be reduced in cancer patients receiving acetaminophen, although the pain is not controllable. Further studies are required to explore the reasons for the low MEDD in cancer patients receiving acetaminophen.

### Strengths and weaknesses/limitations of the study

The main strength of our research was the long study period, from 2016 to 2020, with a large sample size of cancer patients. Additionally, both weak and strong potency opioids were examined in this study.

This study had several limitations. First, this was a retrospective review that collected data from medical records, thus instances of incorrect data entry were probable because the purpose of the original record entries was only for treatment. Second, although prescriptions in our hospital are and were double-checked by both nurses and pharmacists, incorrect prescription records might still have occured. Third, the dataset used does not have mortality data so our analysis did not cover the mortality of diseases in individual patients. In addition, due to the lack of systematic records of any side effects from prescribed opioids, we were unable to assess tolerance, cases of addiction, or psychological and neurological effects from opioid use. Finally, the MEDD in our study was calculated from the doctors’ opioid prescriptions, which reflected the doses recommended by the doctors, and we had no way to determine the patients’ compliance with the prescriptions.

In conclusion, opioid use in the study university hospital in Southeast Asia was still lower than the global recommended dosage limits. Nonetheless, this study provides useful details on various factors associated with MEDD in this region, including age, cancer duration, cancer stage and metastasis. To improve the underusage situation, a policy to promote national health education on safe and effective opioid usage taking into account these factors is necessary. Proper education will help decrease the fear of opioid use in both healthcare providers and the general population and increase the attention paid to opioid usage. It is necessary to study the clinical use of opioids in tertiary and primary and secondary care hospitals so that cancer patients in Thailand can receive equal opioid therapy anywhere in the country. To better assess national opioid use, future studies should focus on analyzing national databases, such as those of the Thai National Health Security Office.

## Data availability statement

The datasets presented in this article are not readily available because the dataset contains patients’ identification and personal information. Requests to access the datasets should be directed to orapan.f@psu.ac.th.

## Ethics statement

The studies involving human participants were reviewed and approved by the Office of Human Research and Ethics Committee, Faculty of Medicine, Prince of Songkla university (approval no. REC.64-278-9-1). Written informed consent for participation was not required for this study in accordance with the national legislation and the institutional requirements.

## Author contributions

TI supervised the study procedure. PL analyzed and interpreted the data and wrote the manuscript. OF and TI reviewed and revised the manuscript. All authors contributed to the article and approved the submitted version
