# Supplementary material for: The trend of opioid prescriptions among cancer patients in a tertiary hospital: A multimethod quantitative study
Source: Front Oncol. 2023 Apr 11;13:1138169. doi: 10.3389/fonc.2023.1138169 (PMC10128996; doi:10.3389/fonc.2023.1138169)
Supplement: Supplementary file 1 [file DataSheet_1.docx]

**Supplementary Materials**

Supplementary Table 1: Morphine Equivalent Daily Dose (MEDD) from Multiple Linear Regression with GEE* for Patients Receiving Concurrent Drug(s).

| Concurrent Drug(s) | | Univariable | | Multivariable | |  |
| --- | --- | --- | --- | --- | --- | --- |
|  |  | MEDD diff (mg/d) | p-value | MEDD diff  (mg/d) | p-value | |
| Paracetamol | No | ref | <0.001 | ref | <0.001 | |
|  | Yes | -4.02 (-4.75–-3.28) |  | -3.75 (-4.47–-3.03) |  | |
| Anticonvulsant | No | ref | <0.001 | ref | <0.001 | |
|  | Yes | 8.34 (6.50–10.18) |  | 8.38 (6.54–10.22) |  | |
| Corticosteroid | No | Ref | 0.001 | Ref | 0.142 | |
|  | Yes | 1.38 (0.57–2.19) |  | 0.60 (-0.20–1.40) |  | |
| Antidepressant | No | ref | <0.001 |  | <0.001 | |
|  | Yes | 3.39 (2.56–4.22) |  | 3.17 (2.34–3.99) |  | |

*GEE = Generalized Estimating Equation

Supplementary Table 2: Morphine Equivalent Daily Dose (MEDD) by Cancer Characteristics divided by Sex

|  | **Total Number of Patients** | | **Female** | | **Male** | |
| --- | --- | --- | --- | --- | --- | --- |
| **Characteristic** | **Number of Patients (%)** | **MEDD (Mean ±SD)** | **Number of Patients (%)** | **Characteristics** | **Number of Patients (%)** | **MEDD (Mean ±SD)** |
| Primary Cancer Site |  |  |  |  |  |  |
| Bones and articular cartilage | 49 (0.7) | 36.5±39.0 | 23 (0.7) | 32.3±33.8 | 26 (0.6) | 38.1±42.4 |
| Breast | 531 (7.2) | 25.1±18.8 | 529 (16.5) | 25.1±18.8 | 2 (0.0) | 22.3±10.1 |
| Endocrine system | 80 (1.8) | 26.0±14.4 | 46 (1.4) | 24.8±16.2 | 34 (0.8) | 26.9±12.6 |
| Eye, brain, and other nervous system | 45 (0.7) | 18.6±7.4 | 21 (0.7) | 18.5±8.3 | 24 (0.6) | 18.8±6.7 |
| Gastrointestinal | 2,112 (28.4) | 26.4±23.4 | 719 (22.5) | 27.1±27.8 | 1,393 (33.0) | 26.1±20.9 |
| Gynecological | 767 (10.3) | 25.7±18.6 | 767 (24.0) | 25.7±18.6 | 0 (0.0) | - |
| Haematological | 320 (4.3) | 23.3±26.9 | 146 (4.6) | 19.6±13.6 | 174 (4.1) | 25.8±32.9 |
| Head and neck | 1,369 (18.7) | 31.7±19.8 | 321 (10.0) | 31.8±19.9 | 1,048 (24.8) | 31.7±19.8 |
| Male genitals | 231 (3.1) | 22.4±15.1 | 0 (0) | - | 231 (5.5) | 22.4±15.1 |
| Malignant neoplasms of ill-defined, secondary, and unspecified sites | 112 (1.5) | 31.5±21.5 | 29 (0.9) | 33.7±25.6 | 83 (2.0) | 30.9±20.1 |
| Mesothelial and soft tissue | 141 (1.9) | 30.3±25.5 | 85 (2.7) | 27.8±14.2 | 56 (1.3) | 34.1±36.7 |
| Respiratory and intrathoracic organs | 1,344 (18.1) | 28.5±23.0 | 404 (12.6) | 28.3±27.0 | 940 (22.3) | 28.5±20.6 |
| Skin | 130 (1.8) | 26.4±17.8 | 63 (2.0) | 25.2±16.0 | 67 (1.6) | 27.8±19.8 |
| Urinary tract | 193 (2.6) | 26.5±21.6 | 47 (1.5) | 27.0±23.5 | 146 (3.5) | 27.7±21.0 |
| Stage |  |  |  |  |  |  |
| 1 | 282 (3.8) | 24.4±23.6 | 187 (7.0) | 24.8±27.6 | 95 (2.9) | 23.5±13.0 |
| 2 | 716 (9.6) | 24.1±17.4 | 446 (16.7) | 22.9±16.2 | 270 (8.2) | 25.8±18.9 |
| 3 | 1,481 (20.0) | 26.6±19.7 | 797 (29.9) | 25.3±18.8 | 684 (20.8) | 27.7±20.4 |
| 4 | 3,477 (46.8) | 30.1±23.3 | 1,238 (46.4) | 30.0±25.8 | 2,239 (68.1) | 30.2±21.9 |

* MEDD= Morphine Equivalent Daily Dose
